# Supplementary material for: Epidemiological Profile of Hospitalized Patients with Cystic Fibrosis in Brazil Due to Severe Acute Respiratory Infection during the COVID-19 Pandemic and a Systematic Review of Worldwide COVID-19 in Those with Cystic Fibrosis
Source: Healthcare (Basel). 2023 Jul 4;11(13):1936. doi: 10.3390/healthcare11131936 (PMC10341451; doi:10.3390/healthcare11131936)
Supplement: Supplementary file 1 [file healthcare-11-01936-s001.zip › healthcare-2320587-supplementary.pdf]

Title: Epidemiological Profile of Hospitalized Patients with Cystic Fibrosis in Brazil due to Severe Acute Respiratory Infection During the COVID-19 Pandemic and a Systematic Review of Worldwide COVID-19 in Those with Cystic Fibrosis

Systematic Review Search

"Search: (((COVID-19 OR COVID OR Coronavirus Disease OR Coronavirus Disease 2019 OR SARS-CoV OR SARS-CoV-2 OR SARS OR severe acute respiratory syndrome) AND (Cystic Fibrosis OR CFTR OR Cystic Fibrosis Transmembrane Regulator))) AND (("2019/11/17"[Date - Publication] : "2022/03/25"[Date - Publication])) ("covid 19"[All Fields] OR "covid 19"[MeSH Terms] OR "covid 19 vaccines"[All Fields] OR "covid 19 vaccines"[MeSH Terms] OR "covid 19 serotherapy"[All Fields] OR "covid 19 serotherapy"[Supplementary Concept] OR "covid 19 nucleic acid testing"[All Fields] OR "covid 19 nucleic acid testing"[MeSH Terms] OR "covid 19 serological testing"[All Fields] OR "covid 19 serological testing"[MeSH Terms] OR "covid 19 testing"[All Fields] OR "covid 19 testing"[MeSH Terms] OR "sars cov 2"[All Fields] OR "sars cov 2"[MeSH Terms] OR "severe acute respiratory syndrome coronavirus 2"[All Fields] OR "ncov"[All Fields] OR "2019 ncov"[All Fields] OR (("coronavirus"[MeSH Terms] OR "coronavirus"[All Fields] OR "cov"[All Fields]) AND 2019/11/01:3000/12/31[Date - Publication]) OR ("sars cov 2"[MeSH Terms] OR "sars cov 2"[All Fields] OR "covid"[All Fields] OR "covid 19"[MeSH Terms] OR "covid 19"[All Fields]) OR (("coronavirus"[MeSH Terms] OR "coronavirus"[All Fields] OR "coronaviruses"[All Fields]) AND ("disease"[MeSH Terms] OR "disease"[All Fields] OR "diseases"[All Fields] OR "disease s"[All Fields] OR "diseased"[All Fields])) OR ("covid 19"[MeSH Terms] OR "covid 19"[All Fields] OR "coronavirus disease 2019"[All Fields]) OR ("sars virus"[MeSH Terms] OR ("SARS"[All Fields] AND "virus"[All Fields]) OR "sars virus"[All Fields] OR ("SARS"[All Fields] AND "cov"[All Fields]) OR "sars cov"[All Fields]) OR ("sars cov 2"[MeSH Terms] OR "sars cov 2"[All Fields] OR "sars cov 2"[All Fields]) OR "SARS"[All Fields] OR ("severe acute respiratory syndrome"[MeSH Terms] OR ("severe"[All Fields] AND "acute"[All Fields] AND "respiratory"[All Fields] AND "syndrome"[All Fields]) OR "severe acute respiratory syndrome"[All Fields])) AND ("cystic fibrosis"[MeSH Terms] OR ("cystic"[All Fields] AND "fibrosis"[All Fields]) OR "cystic fibrosis"[All Fields] OR "CFTR"[All Fields] OR (("cystic fibrosis"[MeSH Terms] OR ("cystic"[All Fields] AND "fibrosis"[All Fields]) OR "cystic fibrosis"[All Fields]) AND ("transmembranal"[All Fields] OR "transmembrane"[All Fields] OR "transmembraneous"[All Fields] OR "transmembranes"[All Fields] OR "transmembranous"[All Fields]) AND ("legislation and jurisprudence"[MeSH Subheading] OR ("legislation"[All Fields] AND "jurisprudence"[All Fields]) OR "legislation and jurisprudence"[All Fields] OR "regulations"[All Fields] OR "social control, formal"[MeSH Terms] OR ("social"[All Fields] AND "control"[All Fields] AND "formal"[All Fields]) OR "formal social control"[All Fields] OR "regulate"[All Fields] OR "regulates"[All Fields] OR "regulating"[All Fields] OR "regulation s"[All Fields] OR "regulative"[All Fields] OR "regulator"[All Fields] OR "regulator s"[All Fields] OR "regulators"[All Fields] OR "regulated"[All Fields] OR "regulation"[All Fields]))) AND 2019/11/17:2022/03/25[Date - Publication] Translations COVID-19: ("COVID-19" OR "COVID-19"[MeSH Terms] OR "COVID-19 Vaccines" OR "COVID-19 Vaccines"[MeSH Terms] OR "COVID-19 serotherapy" OR "COVID-19 serotherapy"[Supplementary Concept] OR "COVID-19 Nucleic Acid Testing" OR "covid-19 nucleic acid testing"[MeSH Terms] OR "COVID-19 Serological Testing" OR "covid-19 serological testing"[MeSH Terms] OR "COVID-19 Testing" OR "covid-19 testing"[MeSH Terms] OR "SARS-CoV-2" OR "sars-cov-2"[MeSH Terms] OR "Severe Acute Respiratory Syndrome Coronavirus 2" OR "NCOV" OR "2019 NCOV" OR (("coronavirus"[MeSH Terms] OR "coronavirus" OR "COV") AND 2019/11/01[PDAT] : 3000/12/31[PDAT])) COVID: "sars-cov-2"[MeSH Terms] OR "sars-cov-2"[All Fields] OR "covid"[All Fields] OR "covid-19"[MeSH Terms] OR "covid-19"[All Fields] Coronavirus: "coronavirus"[MeSH Terms] OR "coronavirus"[All Fields] OR "coronaviruses"[All Fields] Disease: "disease"[MeSH Terms] OR "disease"[All Fields] OR "diseases"[All Fields] OR "disease's"[All Fields] OR "diseased"[All Fields] Coronavirus Disease 2019: "covid-19"[MeSH Terms] OR "covid-19"[All Fields] OR "coronavirus disease 2019"[All Fields] SARS-CoV: "sars virus"[MeSH Terms] OR ("sars"[All Fields] AND "virus"[All Fields]) OR "sars virus"[All Fields] OR ("sars"[All Fields] AND "cov"[All Fields]) OR "sars cov"[All Fields] SARS-CoV-2: "sars-cov-2"[MeSH Terms] OR "sars-cov-2"[All Fields] OR "sars cov 2"[All Fields] severe acute respiratory syndrome: "severe acute respiratory syndrome"[MeSH Terms] OR ("severe"[All Fields] AND "acute"[All Fields] AND "respiratory"[All Fields] AND "syndrome"[All Fields]) OR "severe acute respiratory syndrome"[All Fields] Cystic Fibrosis: "cystic fibrosis"[MeSH Terms] OR ("cystic"[All Fields] AND "fibrosis"[All Fields]) OR "cystic fibrosis"[All Fields] Cystic Fibrosis: "cystic fibrosis"[MeSH Terms] OR ("cystic"[All Fields] AND "fibrosis"[All Fields]) OR "cystic fibrosis"[All Fields] Transmembrane: "transmembranal"[All Fields] OR "transmembrane"[All Fields] OR "transmembraneous"[All Fields] OR "transmembranes"[All Fields] OR "transmembranous"[All Fields] Regulator: "legislation and jurisprudence"[Subheading] OR ("legislation"[All Fields] AND "jurisprudence"[All Fields]) OR "legislation and jurisprudence"[All Fields] OR "regulations"[All Fields] OR "social control, formal"[MeSH Terms] OR ("social"[All Fields] AND "control"[All Fields] AND "formal"[All Fields]) OR "formal social control"[All Fields] OR "regulate"[All Fields] OR "regulates"[All Fields] OR "regulating"[All Fields] OR "regulation's"[All Fields] OR "regulative"[All Fields] OR "regulator"[All Fields] OR "regulator's"[All Fields] OR "regulators"[All Fields] OR "regulated"[All Fields] OR "regulation"[All Fields]".

Supplementary Table S1. Distribution of the hospitalized patients with cystic fibrosis due to severe acute respiratory infection in Brazil during the coronavirus disease, COVID-19 pandemic.

States and the Federal District	Place of Notification (N, %)	Place of Residence (N, %)
Acre	2 (0.5%)	2 (0.5%)
Alagoas	5 (1.2%)	5 (1.2%)
Amazonas	6 (1.4%)	6 (1.4%)
Amapá	1 (0.2%)	1 (0.2%)
Bahia	9 (2.1%)	9 (2.1%)
Ceará	16 (3.8%)	16 (3.8%)
Espírito Santo	4 (0.9%)	4 (0.9%)
Federal District	15 (3.5%)	9 (2.1%)
Goiás	12 (2.8%)	17 (4.0%)
Maranhão	2 (0.5%)	2 (0.5%)
Mato Grosso do Sul	8 (1.9%)	8 (1.9%)
Mato Grosso	5 (1.2%)	5 (1.2%)
Minas Gerais	70 (16.4%)	70 (16.4%)
Pará	1 (0.2%)	1 (0.2%)
Paraíba	7 (1.6%)	7 (1.6%)
Paraná	56 (13.1%)	55 (12.9%)
Pernambuco	10 (2.3%)	10 (2.3%)
Piauí	0 (0.0%)	0 (0.0%)
Rio de Janeiro	15 (3.5%)	16 (3.8%)
Rio Grande do Norte	7 (1.6%)	7 (1.6%)
Rio Grande do Sul	29 (6.8%)	29 (6.8%)
Roraima	0 (0.0%)	0 (0.0%)
Rondônia	1 (0.2%)	1 (0.2%)
Santa Catarina	28 (6.6%)	29 (6.8%)
São Paulo	108 (25.4%)	108 (25.4%)
Sergipe	6 (1.4%)	6 (1.4%)
Tocantins	3 (0.7%)	3 (0.7%)
Total	426	426

We presented the data using the number of individuals (N) and the percentage (%).

Supplementary Table S2. Description of the clinical symptoms and comorbidities of the hospitalized patients with cystic fibrosis according to the severe acute respiratory infection (SARI) groups in Brazil during the coronavirus disease, COVID-19 pandemic.

Markers	Data	SARI due to Another Viral Infection	SARI due to an Unknown Etiological Agent	SARI due to SARS-CoV-2 Infection	Total	*p*
**Clinical symptoms**						
Fever	Yes	19 (82.6%)	168 (58.7%)	72 (61.5%)	259 (60.8%)	0.070 [b]
	No	4 (17.4%)	118 (41.3%)	45 (38.5%)	167 (39.2%)	
Cough	Yes	23 (100.0%)	236 (82.5%)	98 (83.8%)	357 (83.8%)	0.068 [b]
	No	0 (0.0%)	50 (17.5%)	19 (16.2%)	69 (16.2%)	
Sore throat	Yes	0 (0.0%)	19 (6.6%)	14 (12.0%)	33 (7.7%)	0.083 [b]
	No	23 (100.0%)	267 (93.4%)	103 (88.0%)	393 (92.3%)	
Dyspnea	Yes	20 (87.0%)	228 (79.7%)	107 (91.5%)	355 (83.3%)	**0.010** [b]
	No	3 (13.0%)	58 (20.3%)	10 (8.5%)	71 (16.7%)	
Respiratory discomfort	Yes	19 (82.6%)	218 (76.2%)	97 (82.9%)	334 (78.4%)	0.326 [b]
	No	4 (17.4%)	68 (23.8%)	20 (17.1%)	92 (21.6%)	
Peripheral arterial oxygen saturation	<95%	14 (60.9%)	202 (70.6%)	95 (81.2%)	311 (73.0%)	**0.035** [a]
	≥95%	9 (39.1%)	84 (29.4%)	22 (18.8%)	115 (27.0%)	
Diarrhea	Yes	1 (4.3%)	24 (8.4%)	7 (6.0%)	32 (7.5%)	0.685 [b]
	No	22 (95.7%)	262 (91.6%)	110 (94.0%)	394 (92.5%)	
Vomit	Yes	3 (13.0%)	34 (11.9%)	6 (5.1%)	43 (10.1%)	0.071 [b]
	No	20 (87.0%)	252 (88.1%)	111 (94.9%)	383 (89.9%)	
Coryza	Yes	2 (8.7%)	10 (3.5%)	2 (1.7%)	14 (3.3%)	0.209 [b]
	No	21 (91.3%)	276 (96.5%)	115 (98.3%)	412 (96.7%)	
Headache	Yes	0 (0.0%)	12 (4.2%)	9 (7.7%)	21 (4.9%)	0.234 [b]
	No	23 (100.0%)	274 (95.8%)	108 (92.3%)	405 (95.1%)	
Myalgia	Yes	0 (0.0%)	7 (2.4%)	6 (5.1%)	13 (3.1%)	0.324 [b]

	No	23 (100.0%)	279 (97.6%)	111 (94.9%)	413 (96.9%)	
Other clinical symptoms	Yes	3 (13.0%)	37 (12.9%)	18 (15.4%)	58 (13.6%)	0.803 [b]
	No	20 (87.0%)	249 (87.1%)	99 (84.6%)	368 (86.4%)	
**Comorbidities**						
Cardiopathy	Yes	0 (0.0%)	22 (7.7%)	32 (27.4%)	54 (12.7%)	**< 0.001** [b]
	No	23 (100.0%)	264 (92.3%)	85 (72.6%)	372 (87.3%)	
Hematological disease	Yes	0 (0.0%)	0 (0.0%)	2 (1.7%)	2 (0.5%)	0.180 [b]
	No	23 (100.0%)	286 (100.0%)	115 (98.3%)	424 (99.5%)	
Down's syndrome	Yes	0 (0.0%)	3 (1.0%)	0 (0.0%)	3 (0.7%)	0.627 [b]
	No	23 (100.0%)	283 (99.0%)	117 (100.0%)	423 (99.3%)	
Liver disease	Yes	1 (4.3%)	7 (2.4%)	1 (0.9%)	9 (2.1%)	0.299 [b]
	No	22 (95.7%)	279 (97.6%)	116 (99.1%)	417 (97.9%)	
Asthma	Yes	3 (13.0%)	11 (3.8%)	8 (6.8%)	22 (5.2%)	0.082 [b]
	No	20 (87.0%)	275 (96.2%)	109 (93.2%)	404 (94.8%)	
Diabetes mellitus	Yes	0 (0.0%)	29 (10.1%)	17 (14.5%)	46 (10.8%)	0.093 [b]
	No	23 (100.0%)	257 (89.9%)	100 (85.5%)	380 (89.2%)	
Neurological disease	Yes	0 (0.0%)	10 (3.5%)	4 (3.4%)	14 (3.3%)	1.000 [b]
	No	23 (100.0%)	276 (96.5%)	113 (96.6%)	412 (96.7%)	
Immunosuppressive disease	Yes	0 (0.0%)	9 (3.1%)	8 (6.8%)	17 (4.0%)	0.175 [b]
	No	23 (100.0%)	277 (96.9%)	109 (93.2%)	409 (96.0%)	
Kidney disease	Yes	0 (0.0%)	7 (2.4%)	3 (2.6%)	10 (2.3%)	1.000 [b]
	No	23 (100.0%)	279 (97.6%)	114 (97.4%)	416 (97.7%)	
Obesity	Yes	0 (0.0%)	3 (1.0%)	4 (3.4%)	7 (1.6%)	0.225 [b]
	No	23 (100.0%)	283 (99.0%)	113 (96.6%)	419 (98.4%)	

We presented the data using the number of individuals (N) and the percentage (%); SARS-CoV-2, severe acute respiratory syndrome coronavirus 2; [a], we performed the statistical analysis using the chi-square test; [b], we performed the statistical analysis using the Fisher's exact test. We adopted an alpha error of 0.05. We demonstrated the significant *p* using the bold type.

Supplementary Table S3. Association between the need for intensive care unit, the need for mechanical ventilation, and the death of the hospitalized patients with cystic fibrosis according to the severe acute respiratory infection (SARI) groups in Brazil during the coronavirus disease, COVID-19 pandemic.

Markers	Data	SARI due to Another Viral Infection	SARI due to an Unknown Etiological Agent	SARI due to SARS-CoV-2 Infection	Total	*p*
**Intensive care unit**	Yes	1 (4.3%)	82 (28.7%)	52 (44.4%)	135 (31.7%)	**< 0.001** [a]
	No	22 (95.7%)	204 (71.3%)	65 (55.6%)	291 (68.3%)	
**Mechanical ventilatory support**	Invasive	0 (0.0%)	38 (13.3%)	29 (24.8%)	67 (15.7%)	**0.007** [a]
	Non-invasive	15 (65.2%)	166 (58.0%)	63 (53.8%)	244 (57.3%)	
	None	8 (34.8%)	82 (28.7%)	25 (21.4%)	115 (27.0%)	
**Outcomes**	Clinical recovery	23 (100.0%)	231 (80.8%)	65 (55.6%)	319 (74.9%)	**< 0.001** [a]
	Death due to SARI	0 (0.0%)	43 (15.0%)	51 (43.6%)	94 (22.1%)	
	Death was not related to SARI	0 (0.0%)	12 (4.2%)	1 (0.9%)	13 (3.1%)	

We presented the data using the number of individuals (N) and the percentage (%).SARS-CoV-2, severe acute respiratory syndrome coronavirus 2; [a], we performed the statistical analysis using the Fisher's exact test. We adopted an alpha error of 0.05. We demonstrated the significant *p* using the bold type.

Supplementary Table S4. Association between clinical symptoms and comorbidities of the hospitalized patients with cystic fibrosis and SARS-CoV-2 infection in Brazil according to the outcome during the coronavirus disease, COVID-19 pandemic.

Markers	Data	Death due to SARI	Clinical Recovery	Total	*p*
**Clinical Symptoms**					
**Fever**	Yes	25 (49.0%)	47 (72.3%)	72 (62.1%)	**0.013** [a]
	No	26 (51.0%)	18 (27.7%)	44 (37.9%)	
**Cough**	Yes	42 (82.4%)	55 (84.6%)	97 (83.6%)	0.803 [a]
	No	9 (17.6%)	10 (15.4%)	19 (16.4%)	
**Sore throat**	Yes	6 (11.8%)	8 (12.3%)	14 (12.1%)	1.000 [a]
	No	45 (88.2%)	57 (87.7%)	102 (87.9%)	

**Dyspnea**	Yes	49 (96.1%)	57 (87.7%)	106 (91.4%)	0.182 [b]
	No	2 (3.9%)	8 (12.3%)	10 (8.6%)	
**Respiratory discomfort**	Yes	44 (86.3%)	52 (80.0%)	96 (82.8%)	0.461 [a]
	No	7 (13.7%)	13 (20.0%)	20 (17.2%)	
**Peripheral arterial oxygen saturation**	<95%	46 (90.2%)	48 (73.8%)	94 (81.0%)	**0.032** [a]
	≥95%	5 (9.8%)	17 (26.2%)	22 (19.0%)	
**Diarrhea**	Yes	5 (9.8%)	2 (3.1%)	7 (6.0%)	0.238 [b]
	No	46 (90.2%)	63 (96.9%)	109 (94.0%)	
**Vomit**	Yes	2 (3.9%)	4 (6.2%)	6 (5.2%)	0.693 [b]
	No	49 (96.1%)	61 (93.8%)	110 (94.8%)	
**Coryza**	Yes	1 (2.0%)	1 (1.5%)	2 (1.7%)	1.000 [b]
	No	50 (98.0%)	64 (98.5%)	114 (98.3%)	
**Headache**	Yes	4 (7.8%)	5 (7.7%)	9 (7.8%)	1.000 [b]
	No	47 (92.2%)	60 (92.3%)	107 (92.2%)	
**Myalgia**	Yes	4 (7.8%)	2 (3.1%)	6 (5.2%)	0.403 [b]
	No	47 (92.2%)	63 (96.9%)	110 (94.8%)	
**Other symptoms**	Yes	5 (9.8%)	13 (20.0%)	18 (15.5%)	0.196 [a]
	No	46 (90.2%)	52 (80.0%)	98 (84.5%)	
**Comorbidities**					
**Cardiopathy**	Yes	15 (29.4%)	17 (26.2%)	32 (27.6%)	0.835 [a]
	No	36 (70.6%)	48 (73.8%)	84 (72.4%)	
**Hematological disease**	Yes	1 (2.0%)	1 (1.5%)	2 (1.7%)	1.000 [b]
	No	50 (98.0%)	64 (98.5%)	114 (98.3%)	
**Liver disease**	Yes	1 (2.0%)	0 (0.0%)	1 (0.9%)	0.440 [b]
	No	50 (98.0%)	65 (100.0%)	115 (99.1%)	
**Asthma**	Yes	5 (9.8%)	3 (4.6%)	8 (6.9%)	0.297 [b]
	No	46 (90.2%)	62 (95.4%)	108 (93.1%)	
**Diabetes mellitus**	Yes	6 (11.8%)	11 (16.9%)	17 (14.7%)	0.598 [a]
	No	45 (88.2%)	54 (83.1%)	99 (85.3%)	
**Neurological disease**	Yes	1 (2.0%)	3 (4.6%)	4 (3.4%)	0.630 [b]
	No	50 (98.0%)	62 (95.4%)	112 (96.6%)	
**Immunosuppressive disease**	Yes	4 (7.8%)	4 (6.2%)	8 (6.9%)	0.729 [b]
	No	47 (92.2%)	61 (93.8%)	108 (93.1%)	
**Kidney disease**	Yes	1 (2.0%)	2 (3.1%)	3 (2.6%)	1.000 [b]
	No	50 (98.0%)	63 (96.9%)	113 (97.4%)	
**Obesity**	Yes	3 (5.9%)	1 (1.5%)	4 (3.4%)	0.319 [b]
	No	48 (94.1%)	64 (98.5%)	112 (96.6%)	

No patient presented with Down syndrome; We presented the data using the number of individuals (N) and the percentage (%); SARI, severe acute respiratory infection; SARS-CoV-2, severe acute respiratory syndrome coronavirus 2; [a], we performed the statistical analysis using the chi-square test; [b], we performed the statistical analysis using the Fisher's exact test. We adopted an alpha error of 0.05. We demonstrated the significant *p* using the bold type.

Supplementary Table S5. Association between the need for intensive care unit and mechanical ventilatory support with risk of death in hospitalized patients with cystic fibrosis and SARS-CoV-2 infection in Brazil during the coronavirus disease, COVID-19 pandemic.

Markers	Data	Death due to SARI	Clinical Recovery	Total	*p*
**Intensive care unit**	Yes	34 (66.7%)	17 (26.2%)	51 (44.0%)	**< 0.001** [a]
	No	17 (33.3%)	48 (73.8%)	65 (56.0%)	
**Mechanical ventilatory support**	Invasive	27 (52.9%)	2 (3.1%)	29 (25.0%)	**< 0.001** [b]
	Non-invasive	21 (41.2%)	41 (63.1%)	62 (53.4%)	
	No	3 (5.9%)	22 (33.8%)	25 (21.6%)	

We presented the data using the number of individuals (N) and the percentage (%); SARI, severe acute respiratory infection; SARS-CoV-2, severe acute respiratory syndrome coronavirus 2; [a], we performed the statistical analysis using the chi-square test; [b], we performed the statistical analysis using the Fisher's exact test. We adopted an alpha error of 0.05. We demonstrated the significant *p* using the bold type.
